# The hip in arthrogryposis

**DOI:** 10.1007/s11832-015-0693-5

**Published:** 2015-10-19

**Authors:** Christopher Bradish

**Affiliations:** Orthopaedic and Spinal Surgery Unit, Great Ormond Street Hospital, Great Ormond Street, London, WC1N 3JH UK

**Keywords:** Arthrogryposis, Amyoplasia, Hip, Dislocation, MAOR

## Abstract

**Pathology:**

Hip dislocation is seen in approximately 30 % of children with amyoplasia and approximately 50 % of these will be bilateral.

**Treatment:**

Closed reduction is rarely successful. Open reduction is indicated for unilateral dislocations and for the majority of bilateral dislocations. Reduction is recommended via a medial approach.

**Results:**

A long-term satisfactory outcome can be achieved but with some loss of hip range of movement.

## Introduction

The aim in arthrogryposis should always be to maximise the potential of the child. In the child with amyoplasia there may be significant limb deformities and muscle weakness and it is sometimes difficult initially to determine whether the child has walking potential. However, whether the child is ultimately going to be a walker or to rely on a wheelchair, it is beneficial to have a level pelvis to aid sitting and standing. It is generally agreed that unilateral dislocations in children with amyoplasia should be reduced. There remains some debate, however, with regards to the management of children with bilaterally dislocated hips.

## Hip deformities in amyoplasia

The child with amyoplasia may be born with normal hips, but the majority will have contractures, with some having unilateral or bilateral dislocation. Classically, the hips are abducted, flexed and externally rotated. The approximate incidence of deformities in amyoplasia was reported by Staheli et al. from Seattle [1]. Amongst a group of 95 children with amyoplasia, they found the following deformities:

- Only 6 upper limb deformities
- Lower limb involvement in 89 children
- Hip dislocation in 26 children, 40 dislocations
- Unilateral dislocation in 12
- Bilateral dislocation in 14

In addition, they reported flexion contractures in almost 40 % and external rotation or abduction contractures in 20 %.

## Management of hip deformities in AMC

### Hip flexion contracture

The hips may be reduced but with contractures. A hip flexion contracture is often compensated for by increased lumbar lordosis. Minor flexion contractures frequently resolve, but a persistent contracture above 45° will usually require release. It is best to delay the release of a flexion contracture until it is clear that the child is going to achieve walking. Hoffer et al. [2], in their paper reporting the ambulatory status of children with AMC, found that the maximum hip flexion contracture compatible with walking was 30°. They also reported that in order to achieve walking, children required hip extensor power of 4 or greater, quadriceps strength of at least power 3, or crutchable upper extremities and orthotic substitutes.

#### Technique of flexion contracture release

A flexion contracture can be released by an anterior approach to the hip with release of sartorius, rectus femoris and the anterior hip capsule as necessary. The patient is draped so that a Thomas test can be performed while sequentially releasing the contractures, and release should be performed to reduce the contracture to 10–20°. Bilateral contractures can be released at the same time. Postoperatively, the child is put into a hip spica for 2 weeks and then the spica can be bivalved and used as a night splint for an additional 6–8 weeks.

### External rotation deformity

An external rotation deformity is frequently seen with hip external rotation but limited internal rotation. This is due to femoral retroversion, which will spontaneously improve with growth; surgical correction, which would require an internal rotation femoral osteotomy, is not required.

It should be noted that the child may walk with the foot pointing forwards, as the internally rotated clubfoot compensates for the hip deformity. This can put a strain on the knee, with stretching of the collateral ligaments.

### Abduction contracture

The child born with an abduction contracture will have the “buddha-like” position of abduction–external rotation. These hips will normally be reduced and the main problem is bringing the thighs together for sitting. Clinically there is an excess of soft tissue in the groin and the inguinal crease may be displaced to mid-thigh level. The hip abductors may be contracted. Significant spontaneous improvement can be anticipated, and this is aided by physiotherapy and parental stretching exercises. Staheli has reported that rarely the medial soft tissue may require excision to improve adduction and appearance, while Hoffer reported a requirement for femoral osteotomies to achieve adduction in four nonambulatory patients in his series.

### Hip dislocation

Hip dislocation is present in approximately 30 % of children with amyoplasia, and approximately 50 % of these will be bilateral. It is appropriate to reduce a unilateral dislocation in most cases, not only to facilitate mobility but also to limit pelvic obliquity and the development of scoliosis. The treatment of the bilateral dislocation is more controversial and many authors have favoured leaving bilateral dislocations untreated. Lloyd Roberts and Lettin reported in 1970 [3] the Great Ormond Street (London) experience of treating 52 children with arthrogryposis; amongst these were 13 children with dislocated hips, ten unilateral and three bilateral. They advocated reduction of unilateral dislocations but stated “we hesitate to reduce the dislocation only in those with rare bilateral dislocations in which rigidity ensures stability while at the same time making it improbable that useful movement will be achieved even if the hips are reduced”. They did note however that “these six hips developed an obstinate fixed flexion deformity”. This perception held sway until the work of Staheli demonstrated that it was possible, with a medial approach open reduction, to reduce bilateral dislocations with a satisfactory outcome, and that both hips could be reduced at the same operative procedure.

Hip dislocation in amyoplasia is congenital and teratologic. Although reduction may in some cases be possible by closed means, in the majority this is impossible and an open reduction is required. At surgery, one finds a shortened iliopsoas tendon and a contracted joint capsule.

The surgical technique for reduction can be via an anterior approach or a medial approach open reduction (MAOR). The MAOR was first popularised for the hip in amyoplasia by Staheli, and in his 1987 paper [4] he compared the results of using the MAOR with an anterolateral approach.

Eighteen patients with 24 dislocated hips were treated (12 unilateral and six bilateral). A closed reduction was achieved in four patients (two unilateral dislocations and two bilateral) and the remaining 14 patients (ten unilateral and four bilateral) were treated surgically. An anterolateral approach was used in five patients (all with unilateral dislocations) and the MAOR in nine patients (five unilateral dislocations and four bilateral). The anterolateral group were operated on at a mean age of 18 months (range 6–34 months) and the MAOR group at a mean age of 9 months (range 2–27 months). The patients were comparable in terms of the severity of their arthrogryposis and involvement of other joints. Follow-up was from 9 to 245 months (mean 73 months), and the results were rated on the basis of assessment involving maintenance of reduction, range of motion, function and complications, as detailed below:

**Table:** 

Rating	Hip reduction	Range of motion	Function	Complications
Good	Concentric	Unchanged	No limitation	None
Fair	Subluxed	≤20° loss	Minimal limitation	Minor
Poor	Dislocated	>20° loss	Limits mobility	Serious

Using these criteria, the ratings of the 24 hips at follow-up were as shown below:

**Table:** 

Treatment	Good	Fair	Poor
Closed reduction	4	2	0
Open reduction			
Anterolateral	2	1	2
MAOR	12	1	0

They concluded that patients had a better ROM and function following hip reduction by MAOR, and overall 92 % of the MAOR-treated hips were rated good, as opposed to 40 % treated by an anterolateral approach. With regards to the four bilateral cases treated by MAOR, all remained reduced, two patients were ambulators, one was a good knee walker and one relied on a wheelchair because of other physical and mental impairments. On the basis of these results, Staheli recommended the MAOR for treatment of hip dislocation in arthrogryposis.

His group published a larger series in 1996 [1], reporting the results of using the MAOR on 16 patients, seven with unilateral dislocations and nine with bilateral dislocations. Results were classified as previously detailed, and he reported 80 % good, 12 % fair and 8 % poor results.

Complications reported were one early redislocation diagnosed 1 week after the removal of a 6-week-long spica cast on the left hip in a bilateral MAOR. This was treated by an anterolateral approach open-reduction reoperation, and resulted in stable repositioning and subsequently good hip development and movement. There were two subluxations; one unilateral case was treated by the shelf procedure. One posttreatment dysplasia remained severe whereas four were mild. There were four with avascular necrosis of the proximal femoral epiphysis, three Tönnis grade 1 and one Tönnis grade 2, and these changes tended to regress with time. They did not find any Tönnis grade 3.

In this larger series, the nine patients treated by MAOR for bilateral hip dislocation had a good hip ROM. They did not find a stiff hip in this group, and the acetabular development was satisfactory. Among the bilateral cases treated by MAOR, the occurrence of avascular necrosis was only 11 %. These were not more severe than Tönnis grade 2 and tended to regress spontaneously with time. Pelvic obliquity and pain in the hip did not occur after bilateral MAOR.

Although Staheli has demonstrated that the MAOR can achieve a satisfactory reduction with bilateral dislocations, there remains controversy about the appropriateness of reducing bilateral dislocations. As the children with dislocated hips can walk and have reasonable mobility and little pain, proponents of accepting dislocations believe reduction is unnecessary. In contrast, Staheli advocates that reduction improves the quality of gait in both function and appearance [5]. The hips are more stable and the gait is more efficient. This was reiterated by Yau [6], who stated, “persistent dislocation of the hip is associated with poor long term results”. The author shares this view and recommends reduction for both unilateral and bilateral dislocations.

#### Technique of MAOR

The MAOR can be used in children with amyoplasia from 3 months of age onwards, and it is possible to combine this surgery with surgery to the knee and feet.

The patient is draped to allow a wide exposure should knee and foot procedures be required at the same time. If multiple procedures are being performed, the hip is operated on last. An arthrogram is performed; this not only defines the anatomy of the hip but also distends the hip capsule to aid dissection. A groin incision is used, and I favour division of the tendon of adductor longus to aid the access to the hip. Finger dissection is then used to define the interval between adductor longus and pectineus. The lesser trochanter is palpated, and this is aided by external rotation and gentle traction on the limb. The iliopsoas tendon is then identified attaching to the lesser trochanter, with its overlying fat and bursa. The tendon is divided and it then retracts, exposing the hip capsule. After that, a Hohmann retractor can be passed superior to the capsule to help bring the hip into the operating field. The joint capsule is then divided with an anterior capsulotomy which is extended medially to include division of the transverse acetabular ligament. The ligamentum teres is excised and any pulvinar fat removed with a rongeur. The hip should now be reducible. A probe is passed behind the femoral head to try and ensure that the limbus is everted. The wound is then closed. Next, if required, surgery on the other hip is undertaken. Screening is used to ensure that the hips are reduced. Finally, a hip spica is applied with the hips being held in the reduced position, and further imaging is taken to ensure reduction.

The hip spica is maintained for 6 weeks. If additional surgery has been undertaken, the foot piece of the spica can be removed at 2 weeks to enable continued manipulation/casting of the feet.

Children are then observed with X-rays at 3, 6 and 12 months, and then with annual X-rays for 3 years and then 3-yearly.

#### The extended anterolateral approach

An alternative approach described for both unilateral and bilateral dislocations is the extended anterolateral approach [7]. In this approach, a transverse incision is made from the medial border of the sartorius to the greater trochanter 3 cm distal to the anterior superior iliac spine. The fascia lata and the tensor fascia lata are transversely dissected at the level of the incision. Gluteus medius and minimus are temporarily detached from their insertions. The rectus femoris, with its reflected head, is exposed and the edges of the head are clearly defined by blunt dissection. The straight head of rectus femoris is then detached from the anterior inferior iliac spine. The psoas tendon is also detached from the lesser trochanter and later transferred to the anterolateral surface of the proximal femur. Tendons of the short external rotators are transected at their insertions. At this point, the ascending branch of the medial femoral circumflex artery is protected. The joint capsule is incised circumferentially near the acetabular rim. The transverse acetabular ligament is divided. The ligamentum teres and pulvinar fat are removed. The redundant parts of the capsule are resected. Capsulorrhaphy is unnecessary. While the hip is held in slight flexion, full internal rotation and 30° of abduction, a complete reduction is confirmed by screening. The transected and detached muscles are reattached except for the short external rotators. A hip spica cast is then applied in the above position for 8 weeks.

They reported the results of reduction of bilateral dislocations using this technique in five children. Surgery was undertaken at a mean age of 31.5 months (range 17–64 months) and the children were followed up for a mean of 11.8 years (range 3.8–19.5 years). Two of the patients required additional surgery: in one a Salter osteotomy on one side and a contralateral varus derotation osteotomy, and in the other a bilateral femoral shortening because of high dislocation. At the final follow-up all children walked without crutches or canes. Two managed independently, one required a long leg brace and two had short leg braces because of knee and/or foot problems. The clinical results were good in eight hips and fair in two, and seven hips were rated as good (group I or II) on the Severin classification.

These children were operated on at an older age than those reported by Staheli, and this procedure has a role in the older child where treatment has been delayed. If surgery is undertaken in the young infant, femoral shortening is not usually required.

#### Additional procedures

Hip dysplasia may be seen in the arthrogrypotic child. This will improve following hip reduction, and migration of hips is infrequently seen because of the associated hip stiffness. Treatment is rarely required, and treatment can be delayed until puberty if the hips are stable. Surgery and postoperative immobilisation should be kept to a minimum to avoid hip stiffness. Staheli has advocated a shelf procedure to augment acetabular cover. Both hips, if needed, can be operated on at the same time, and postoperative immobilisation is limited to 6 weeks in a cast. If a femoral osteotomy is undertaken with secure internal fixation, immobilisation can be avoided.

Despite the surgeon’s best endeavours, there are always some unsatisfactory results, and the author has treated one girl who ended up with both hips ankylosed in extension following open reduction procedures and pelvic osteotomies. In order to salvage her hips to permit sitting, bilateral proximal femoral resections had to be performed.

#### Outcome

There a few long-term studies of children with arthrogryposis. Yau et al. [6] have reported a group of 19 patients with arthrogryposis followed up for a mean of 20 years (range 6–36 years). From an initial cohort of 81 patients with arthrogryposis, 25 (30 %) had hip problems, six of these were lost to follow-up, leaving 19 patients, amongst whom 13 hips were dislocated, nine subluxed and 16 had contractures. Various surgical strategies were utilized. Of the dislocated hips, nine were unilateral and two bilateral. All except one bilateral case were reduced. Closed reduction was tried in three patients but failed in all cases. The anterolateral approach was subsequently used for reduction. This was combined with a Salter osteotomy in four cases and a femoral osteotomy in five. The nine subluxed hips were managed by closed reduction, combined with varus femoral osteotomy in four cases. The 16 contracture patients were treated surgically in nine cases, with soft-tissue releases in six (which had to be repeated in three) and femoral osteotomies in three cases.

At review, 14 patients (73.7 %) were community walkers, 2 (10.5 %) were household walkers and 3 (15.8 %) were wheelchair users. They were able to draw the following conclusions:

1. All patients had a degree of hip flexion contracture, but in the majority this was <30° and did not interfere with walking.
2. There was restriction of movement in all operated hips, with the smallest total movement arc seen in the dislocated group (170 ± 67.3°), but this was not statistically different from the subluxation group (209°) or the contracture group (194°).
3. For patients with a unilateral dislocation treated surgically, the operated hip was stiffer than the non-operated hip but had similar function.
4. The majority of patients reported either no pain or only occasional pain. The subluxation group had least pain.
5. There was a significant correlation between younger age at surgery and total arc of hip movement in the contracture group.
6. They had two patients with bilaterally dislocated hips: the patient treated with open reduction remained a community walker and had significantly greater hip function than the non-operated patient, who was confined to a wheelchair.

## Conclusions

1. Hip dislocation is seen in approximately 30 % of children with amyoplasia, and approximately 50 % of these will be bilateral.
2. Closed reduction is rarely successful for these teratologic dislocations.
3. Open reduction is indicated for unilateral dislocations, and for the majority of bilateral dislocations.
4. Open reduction can be via an anterolateral approach or MAOR. The MAOR has the advantage of being able to be performed bilaterally at the same procedure, and may result in a greater range of movement.
5. Hip capsulorrhaphy is not required with AMC.
6. The treatment of residual dysplasia can be delayed until adolescence.
7. A long-term satisfactory outcome can be achieved following open reduction, albeit with some decrease in the range of hip movement.
